# Serum Retinol-Binding Protein 4 as a Marker for Cardiovascular Disease in Women

**DOI:** 10.1371/journal.pone.0048612

**Published:** 2012-10-31

**Authors:** Khalid M. Alkharfy, Nasser M. Al-Daghri, Paul M. Vanhoutte, Soundararajan Krishnaswamy, Aimin Xu

**Affiliations:** 1 Department of Clinical Pharmacy, College of Pharmacy, King Saud University, Riyadh, Saudi Arabia; 2 Biochemistry Department, College of Science, King Saud University, Riyadh, Saudi Arabia; 3 Biomarkers Research Program, College of Science, King Saud University, Riyadh, Saudi Arabia; 4 Department of Pharmacology and Pharmacy, Faculty of Medicine, The University of Hong Kong, Hong Kong; 5 Department of Medicine, Faculty of Medicine, The University of Hong Kong, Hong Kong; The University of Hong Kong, Hong Kong

## Abstract

**Background:**

Elevated serum level of retinol-binding protein 4 (RBP4) has been associated with obesity-related co-morbidities including insulin resistance, dyslipidemia and hypertension.

**Objectives:**

The present study examined the relationship between serum level of RBP4 and various risk factors related to cardiovascular disease (CVD) in men and women.

**Methods:**

284 subjects (139 males, 145 females), grouped into healthy (n = 60), obese diabetes (n = 60), non-obese diabetes (n = 60), obese non-diabetes (n = 60) and patients with CVD (n = 44), were assessed for anthropometric and biochemical parameters related to obesity, diabetes and CVD. In addition, serum levels of several adipokines, including fatty acid binding protein 4 (FABP4) and lipocalin 2 (LCN2) and RBP4 were measured using specific immunoassays.

**Results:**

Serum RBP4 level correlated significantly with principal component derived from known risk factors of CVD (β = 0.20±0.06, P = 0.002). Significance of this correlation was limited to women (β = 0.20±0.06, P = 0.002) and it persisted even after adjusting for BMI (β = 0.19±0.06, P = 0.002). Overall (n = 284) serum RBP4 values significantly correlated with FABP4 (R = 0.19, p = 0.001). Serum FABP4 level of CVD subjects was significantly higher than healthy control (P = 0.001) and non-obese diabetes (P = 0.04) groups, but this difference was attributable to differences in BMI. Serum LCN2 level correlated well with RBP4 (R = 0.15, P = 0.008) and FABP4 (R = 0.36, P<0.001), but did not differ significantly between CVD and other groups.

**Conclusions:**

Results of this study indicate a significant correlation between serum RBP4 and various established risk factors for CVD and suggest RBP4 may serve as an independent predictor of CVD in women.

## Introduction

Cardiovascular diseases (CVD) are a principal cause of death worldwide and are linked to obesity and metabolic syndrome. Several adipokines secreted by the increased adipose tissue mass, together with the infiltrating macrophages, have been identified as key components of the ‘adipo-cardiovascular axis’ and are main contributors to the pathogenesis of atherosclerosis and other cardiovascular diseases [1], [2]. Among these, the lipocalin family proteins, FABP4, LCN2 and RBP4, have been identified as adipokines associated with obesity, type 2 diabetes and metabolic syndrome.

FABP4, a lipid-binding chaperone protein for fatty acids, is one of the most abundant proteins secreted by mature adipocytes [3] and also by macrophages [4]. Epidemiological studies in different ethnic groups demonstrate a close association between serum levels of FABP4 and a cluster of obesity-related cardiometabolic risk factors [5]–[9]. In particular, plasma FABP4 levels are positively correlated with measures of endothelial dysfunction [10], coronary atherosclerosis [11] and various types of cardiovascular diseases [9], [12]–[14].

**Figure 1:**
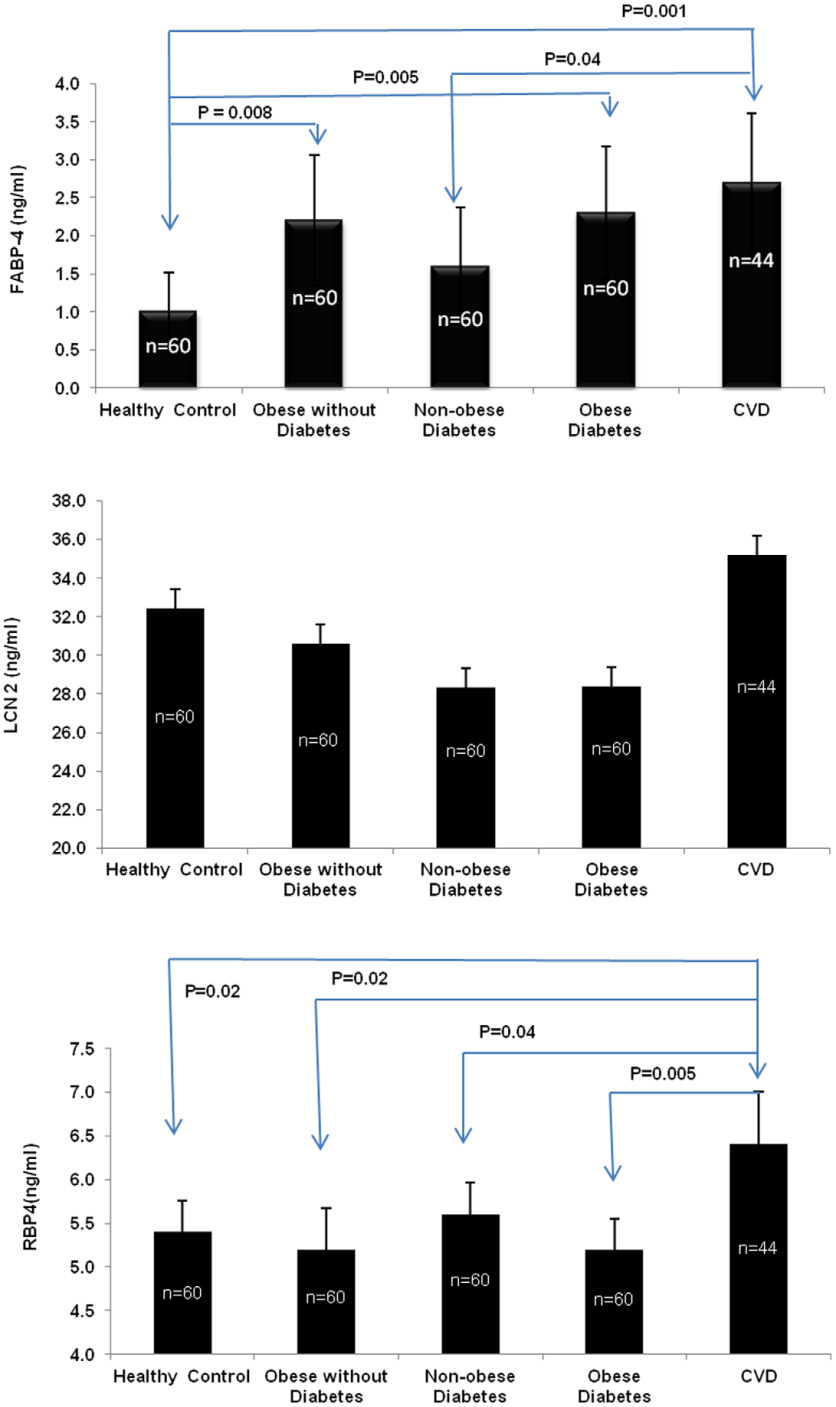
Serum levels of FABP4, LCN2 and RBP4 in obese subjects with or without diabetes and in CVD patients. The CVD group had significantly higher level of serum RBP4 compared to any of the other groups. FABP4 values were significantly higher in obese, obese diabetic and CVD subjects compared to control and non-obese diabetic groups. LCN2 values were higher in CVD subjects than any of the others but this difference did not reach statistical significance (P = 0.46).

LCN2 is a 25 kDa glycoprotein that is secreted abundantly by adipose tissues, and hence elevated in conditions of obesity [15], [16]. Clinical, animal and cellular studies demonstrate its pro-inflammatory properties and its causal involvement in obesity-associated metabolic abnormalities [15]–[18].

RBP4 is the sole carrier of retinol (vitamin A alcohol) in blood and serves to transport it from liver stores to the peripheral tissues. In various chronic diseases associated with obesity RBP4 is produced largely by mature adipocytes [19] and activated macrophages [20] in the adipose tissue. It is an established biomarker of adipose tissue mass and obesity in both children and adults [19]–[21]. This association of RBP4 with body fat has been extended to various obesity-associated metabolic and cardiovascular disorders [19], [22]–[24].

The aim of the present study was to investigate the usefulness of FABP4, LCN2 and RBP4 as biomarkers of CVD by exploring in men and women the association between their serum levels and various known markers related to CVD.

**Table 1:** Clinical and biochemical parameters of study subjects by group.

	Healthy Control	Obese Without Diabetes	Non-obeseDiabetes	Obese Diabetes	CVD	P Value
N	60	60	60	60	44	
M/F	32/28	28/32	27/33	25/35	27/17	0.29
Medications						
Anti-diabetic	-	-	46	55	12	<0.001
Cardiovascular	-	5	7	4	44	<0.001
Smoking status (%)	18.3	11.6	8.3	10.0	16.0	0.46
Age (year)	51.4±13.6	54.3±14.2	58.8±12.3^ab^	52.9±9.5^c^	64.4±9.7^abcd^	<0.001
BMI (kg/m^2^)	22.9±1.6	34.7±3.9^a^	26.5±3.1^ab^	34.0±2.5^ac^	29.5±4.5^abcd^	<0.001
Waist (cm)	82.5±13.4	104.3±16.6	92.2±18.2	101.1±25.3	97.5±20.3	<0.001
Hips (cm)	95.1±15.1	115.6±16.5^a^	97.1±19.2^b^	104.4±26.3^abc^	102.1±22.5^ab^	<0.001
SAD (cm)	19.2±4.4	25.0±7.4^a^	23.0±7.7^a^	25.4±6.0^a^	25.0±10.0^a^	<0.001
Systolic BP (mmHg)	116.5±13.4	128.2±14.6^a^	127.0±14.6^a^	123.8±12.2^a^	128.5±16.3^a^	<0.001
Diastolic BP (mmHg)	74.7±6.6	79.7±7.0^a^	79.3±6.7^a^	78.5±7.4^a^	77.3±7.7^a^	0.002
LDL (mmol/L)	3.4±0.28	3.5±0.27	3.8±0.26^a^	3.4±0.24	3.0±0.32^abcd^	0.006
Triglycerides (mmol/L)	1.5±0.33	1.7±0.28	2.3±0.50^ab^	2.3±0.45^ab^	2.1±0.41^ab^	<0.001
HDL (mmol/L)	1.1±0.38	0.93±0.29	0.88±0.31^a^	0.90±0.38^a^	0.79±0.29^ab^	0.04
Glucose (mmol/L)	4.9±1.2	5.4±1.2	12.2±1.3^ab^	11.8±1.3^ab^	8.7±1.5^abcd^	<0.001
Cholesterol (mmol/L)	5.2±1.2	5.4±1.0	5.9±1.4^ab^	5.6±1.2	5.0±1.5^cd^	0.001
Calcium (mmol/L)	2.5±0.23	2.5±0.21	2.5±0.25	2.6±0.31	2.6±0.38	0.20
Phosphorus (mmol/L)	1.2±0.28	1.3±0.40	1.2±0.30	1.4±0.50	1.2±0.30	0.32

Data represented by mean ± standard deviation;

a indicates group significantly different from control;

b indicates group significantly different from obese without diabetes;

c indicates group significantly different from non-obese diabetes;

d indicates group significantly different from obese diabetes. Level of significance is given at p≤0.05. Abbreviations: BMI, body mass index; SAD, sagittal abdominal diameter; LDL, low-density lipoprotein; HDL, high-density lipoprotein.

## Methodology

### Subjects

A total of 284 subjects, 139 males and 145 females, aged between 51 to 64 years, were selected from the existing Biomarkers Screening in Riyadh Program (RIYADH Cohort), a capital-wide study composed of randomly selected individuals from different Primary Health Care Centers (PHCCs) in Riyadh, Saudi Arabia. The subjects were categorized into healthy control (n = 60), obese without diabetes (n = 60), non-obese diabetes (n = 60), obese diabetes (n = 60) and subjects with history of CVD (n = 44). The CVD subjects had also other conditions including hypertension, dyslipidemia and diabetes, and the treatment medications included both cardiovascular and anti-diabetic drugs (insulin, metformin, statins, aspirin, warfarin, and verapamil). Written and informed consent was obtained individually from all the participants prior to inclusion and commencement of the study. This study was conducted in accordance with the guidelines of the Ethics Committee of the College of Science, King Saud University, by which it was approved.

**Table 2:** Establishment of principal components.

	Rotated Component Loadings	
CDV risk factors	Component 1	Component 2
Age	0.79	−0.11
Cholesterol (mmol/L)	0.31	0.48
HDL (mmol/L)	−0.21	0.90
Systolic BP (mmHg)	0.81	0.14
Eigen value	1.42	1.1
Percentage of variance explained by each component (%)	35.5	26.5
Cumulative percentage	35.5	62.0

Eigen vectors (factor loadings for standardized variables) for first two principal components.

### Clinical and Biochemical Measurements

Clinical and anthropometric parameters, including blood pressure (BP), weight, height, hip and waist circumferences, and sagittal abdominal diameter (SAD) were measured following standard procedures. Body mass index (BMI) was calculated as weight/height^2^ (Kg/m^2^). Fasting blood samples were collected and glucose, triglycerides, total and HDL-cholesterol, calcium and phosphorus levels were measured by chemistry auto-analyzer (Konelab, Espoo, Finland) and concentrations of LDL-cholesterol were calculated using Friedwald’s formula [25].

**Table 3:** Pearson correlations of CVD component 1 and BMI with anthropometric and biochemical parameters in CVD subjects.

	CVD Component 1		BMI		Glucose	
	R	P value	R	P value	R	P value
Age	0.79	<0.001	0.01	0.78	0.11	0.06
BMI (kg/m^2^)	0.05	0.36	1	-	0.07	0.22
Waist (cm)	0.07	0.24	0.48	<0.001	0.05	0.38
Hips (cm)	−0.04	0.55	0.46	<0.001	−0.05	0.42
SAD (cm)	0.17	0.01	0.37	<0.001	0.17	0.007
Systolic BP (mmHg)	0.81	<0.001	0.17	0.005	0.10	0.10
Diastolic BP (mmHg)	0.53	<0.001	0.22	0.001	0.10	0.09
LDL (mmol/L)	0.30	<0.001	−0.06	0.32	0.08	0.14
Triglycerides(mmol/L)	0.25	<0.001	0.006	0.92	0.33	<0.001
HDL (mmol/L)	−0.20	0.002	0.02	0.70	−0.05	0.41
Glucose (mmol/L)	0.15	0.02	0.07	0.22	1	-
Cholesterol(mmol/L)	0.32	<0.001	−0.03	0.65	0.24	<0.001
Calcium (mmol/L)	0.09	0.21	−0.02	0.76	0.20	0.003
Phosphorus (mmol/L)	0.01	0.82	0.16	0.01	0.02	0.79
FABP4 (ng/mL)	0.05	0.43	0.13	0.02	0.02	0.74
LCN2 (ng/mL)	−0.03	0.62	−0.01	0.84	−0.05	0.41
RBP4 (ng/mL)	0.20	0.002	−0.07	0.18	−0.06	0.33

### Measurement of FABP4 in Human Serum

A sandwich immunoassay specific to human FABP4, developed by Antibody and Immunoassay Services (AIS), the University of Hong Kong, was used [13]. A mouse monoclonal antibody specific for human FABP4 was pre-coated onto a microplate. Human serum was diluted (1∶3) into phosphate-buffered saline (PBS) (10 mmol/L sodium phosphate, 137 mmol/L NaCl, 2.7 mmol/L KCl, pH 7.4), and 100 µL of the diluted samples or recombinant standards were applied to each well and incubated at 37°C for one hour. The plates were washed three times and then incubated with 100 µL of biotin-labeled polyclonal antibody specific to human FABP4 for another hour. After being washed three more times with PBS, the wells were incubated with streptavidin-conjugated horseradish peroxidase for one hour and subsequently reacted with tetramethyl-benzidine reagent for 15 minutes. A total of 100 µL of 2 mol/L H_2_SO_4_ was added to each well to stop the reaction, and the absorbance at 450 nm was measured using a micro-plate reader. The inter- and intra- assay variations were 4.5–5.7% and 4.1–6.5%, respectively.

**Table 4:** RBP4 in CVD subjects correlated to component 1.

RBP4 as dependent variable	Parameters	Overall		Female		Male	
		β ± standard error	P value	β± standard error	P value	β± standard error	P value
Model-1	Component 1	0.20±0.06	0.002	0.20±0.06	0.002	0.06±0.12	0.63
Model-2	Component 1	0.22±0.06	0.001	0.19±0.06	0.002	0.09±0.12	0.47
	BMI	−0.08±0.06	0.22	0.03±0.06	0.62	−0.15±0.12	0.21

Linear regression models were used to examine the association of principal component scores with RBP4 levels. Model-1 unadjusted; model-2 adjusted for BMI.

### Measurement of LCN2 in Human Serum

Serum levels of LCN2 were quantified using a monoclonal antibody-based immunoassay developed by AIS as described before [26]. Human serum was diluted (1∶50) into PBS, and 100 µL of the diluted samples or recombinant LCN2 standards were applied to each well of a microplate precoated with a monoclonal antibody specific to LCN2, followed by incubation at 37°C for one hour. The plates were then washed three times and incubated with 100 µL of horseradish peroxidase (HRP)-linked monoclonal antibody specific to human LCN2 for another hour. After another extensive washing step, the wells were incubated with tetramethyl-benzidine reagent for 15 minutes. A total of 100 µL of 2 mol/L H_2_SO_4_ was added to each well to stop the reaction, and the absorbance at 450 nm was measured using a micro-plate reader. Since the increases in absorbance were directly proportional to the amount of captured human LCN2, the sample concentration was calculated from a reference curve. The inter- and intra- assay variations were 5.7–7.6% and 4.8–6.5%, respectively.

**Table 5:** Correlation analysis of anthropometric and clinical parameters with serum RBP4, FABP4 and LCN2.

Overall (N = 284)	RBP4 (ng/mL)		FABP4(ng/mL)		LCN2 (ng/mL)	
	R	p value	R	p value	R	p value
N	284		284		284	
Age (year)	0.15	0.008	0.18	0.002	−0.06	0.27
BMI (kg/m^2^)	−0.10	0.09	0.25	<0.001	−0.01	0.84
Waist (cm)	0.002	0.97	0.21	<0.001	0.08	0.16
Hips(cm)	−0.08	0.16	0.20	0.001	0.03	0.65
SAD (cm)	−0.01	0.81	0.24	<0.001	0.04	0.47
Systolic BP (mmHg)	0.10	0.10	0.15	0.01	−0.06	0.32
Diastolic BP (mmHg)	0.05	0.35	0.18	0.004	0.03	0.62
LDL (mmol/L)	0.02	0.69	−0.12	0.05	0.13	0.03
Triglycerides (mmol/L)	0.28	<0.001	0.05	0.55	0.23	0.03
HDL (mmol/L)	−0.06	0.33	0.04	0.48	−0.13	0.02
Glucose (mmol/L)	−0.03	0.59	0.06	0.27	−0.05	0.40
Cholesterol (mmol/L)	0.10	0.07	−0.07	0.25	−0.02	0.72
Calcium (mmol/L)	0.14	0.04	0.02	0.78	−0.02	0.75
Phosphorus (mmol/L)	0.02	0.75	0.06	0.34	0.14	0.03
FABP4 (ng/mL)	0.19	0.001	-	-	0.36	<0.001
RBP4 (ng/mL)	-	-	-	-	0.15	0.008

### Measurement of RBP4 in Human Serum

Serum RBP4 was measured by a monoclonal antibody-based rapid immunoassay developed by AIS [13]. Human serum was diluted (1∶2000) into phosphate-buffered saline (PBS), and 100 µL of the diluted samples or recombinant RBP4 standards were applied to each well of a microplate precoated with a monoclonal antibody specific to human RBP4, followed by incubation at 37°C for one hour. The plates were then washed three times and incubated with 100 µL of HRP-linked monoclonal antibody specific to human RBP4 for another hour. After extensive washing, the wells were incubated with tetramethyl-benzidine reagent for 15 minutes, followed by the addition of 100 µL of 2 mol/L H_2_SO_4_ to stop the reaction. The absorbance at 450 nm was measured using a micro-plate reader, and RBP4 concentration in the samples calculated from a reference curve. The inter- and intra- assay variations were 5.2–8.1% and 6.3–7.8%, respectively.

### Statistical Analysis

Variables are presented as mean ± standard deviation. All variables were checked for normality. Non-Gaussian variables were either square root or log transformed. Group comparisons were carried out using Analysis of variance (ANOVA) followed by the LSD *post hoc* test. Chi-Square analysis was used to compare categorical variables. Principal component analysis was used to account for the primary risk factors for CVD. Principal component analysis transforms the original variables into a new set of uncorrelated factors (principal components) that account for the maximal proportion of the variance in the data, with each component being a linear combination of the original observed variables [27]–[29]. Principal component 1 was the linear combination of variables that accounted for the largest proportion of variance in the data. Numbers of components were retained based on the following rules: (a) Eigen values of 1 or greater or (b) components above the break in the scree plot. A varimax rotation was used to obtain set interpretable components. The components were interpreted based on the loadings of risk factors. Principal component scores were calculated for each subject. Pearson’s correlation was used to assess variation between variables of interest and component scores. Linear regression models were used to examine the association of principal component scores with RBP4 levels. Three linear models were generated, adjusting for age and BMI for each gender. Statistical analysis was performed using SPSS version 16.0 (Statistical package for Social Science, Inc., Chicago IL, USA), and a level of significance was accepted when p was less than 0.05.

## Results

Since FABP4, LCN2 and RBP4 have been reported to relate to insulin resistance and metabolic syndrome, the serum values of these adipokines were measured in a Saudi population grouped into healthy control, obese without diabetes, non-obese diabetes, obese diabetes and subjects with CVD, to explore their potential as biomarkers for CVD (Figure 1). FABP4 values were significantly higher in obese (2.2±0.86 ng/mL), obese diabetes (2.3±0.87 ng/mL) and CVD subjects (2.7±0.90 ng/mL) compared to control (1.01±0.51 ng/mL) and non-obese diabetes (1.6±0.77 ng/mL) groups. However, BMI adjusted FABP4 values of the healthy control and CVD groups were not significantly different (P = 0.18). Serum LCN2 values did not show any significant difference between these groups. RBP4 values were significantly higher in CVD compared to any of the other groups (P = 0.04).

Mean values of various clinical and biochemical parameters measured in different groups are given in Table 1. CVD group showed significant differences with respect to the following parameters: CVD group had lower BMI values than both the obese groups; LDL of CVD was lower than all the other groups; triglyceride value of CVD was lower than that of healthy control and obese non-diabetes groups.

CVD risk score is a risk assessment tool, developed by National Cholesterol Education Program (NCEP) for estimating 10 year risk of having a heart attack, by including various risk factors like age, gender, total cholesterol, HDL smoking status and systolic blood pressure in the analysis. Principal component analysis (PCA) is a mathematical technique that transforms a number of correlated variables into a reduced number of uncorrelated variables called principal components, of which the first principal component captures the maximal variance [27]–[29]. Hence, the first principal component derived from different risk factors would be an efficient way to quantify the risk of developing CVD.

We performed the principal component analysis, by including all the continuous parameters mentioned above, on subjects of all groups, including CVD. Two principal components were selected based on Eigen values (>1) and scree plots (Table 2). All categorical parameters were analyzed by stratification. Component 1 carries all major risks of developing CVD according to NCEP criteria. Components 1 and 2 explained 35.5% and 26.5% of total variance, giving a cumulative percentage of 62.0%. The highest loaded components were systolic blood pressure (0.81) and age (0.79).

To assess the validity of PCA, Pearson correlation analysis of CVD component 1 with anthropometric and biochemical parameters in CVD subjects was performed (Table 3). In addition to the parameters comprising component 1, established risk factors, including LDL (R = 0.30, p<0.001) and triglyceride (R = 0.25, P<0.001) of CVD group, showed significant association with component 1. However, clinical parameters relevant to CVD, namely, LDL, triglyceride, HDL and total cholesterol did not show any significant association with BMI. Serum levels of FABP4 and LCN2 in CVD subjects did not correlate significantly with component 1. Serum RBP4 values in CVD subjects correlated significantly with component 1 (β = 0.20±0.06, P = 0.002), but not with BMI or glucose levels.

Component 1 values of the CVD subjects were analyzed according to gender (Table 4). The gender adjusted component 1 values showed significant correlation with RBP4 only in women (β = 0.20±0.06, P = 0.002). In female CVD subjects, component 1 values were significantly correlated to RBP4 even after adjusting the data for BMI (β = 0.19±0.06, P = 0.002).

To validate RBP4 as a predictive marker of CVD, correlation analysis of anthropometric and clinical parameters with serum RBP4, FABP4 and LCN2 values of the overall study subjects (n = 284) was performed (Table 5). Correlation analysis of the three measured adipokines revealed that serum FABP4 level correlated significantly with parameters associated with obesity, including BMI (R = 0.25, P = 0.002) and systolic (R = 0.15, P = 0.01) and diastolic (R = 0.18, P = 0.004) blood pressure. On the other hand, LCN2 concentration correlated significantly with LDL (R = 0.13, P = 0.03) and triglyceride (R = 0.23, P = 0.03), and were inversely related to HDL (R = −0.13, P = 0.02). Serum RBP4 values were significantly associated with age (R = 0.15, P = 0.008), triglycerides (R = 0.28, p<0.001), calcium (R = 0.14, P = 0.04), FABP4 (R = 0.19, P = 0.001) and LCN2 (R = 0.15, P = 0.008).

Analysis of the influence of gender on the level of each of these three biomarkers in CVD subjects indicated significant differences only for RBP4 and this difference was seen across all the groups except obese diabetic patients (Table S1).

## Discussion

Data from 284 Saudi subjects, grouped into healthy, obese diabetes, non-obese diabetes, obese non-diabetes and patients with CVD, provide evidence supporting a biomarker role for serum RBP4 for CVD. Serum RBP4 levels of CVD subjects correlated well with ‘component 1′, a measure of CVD risk occurring over the next 10 years, calculated based on different risk factors established by NCEP. In our data, component 1 correlated well with many components of the CVD. Even though RBP4, FABP4 and LCN2 levels correlated significantly with each other in the entire study population (n = 284) and FABP4 levels were significantly higher in CVD subjects than in healthy control, FABP4 levels did not differ significantly from that of obese subjects and LCN2 levels did not differ significantly in CVD compared to healthy control or other groups. Neither component 1 nor RBP4 showed significant association with BMI of CVD subjects, indicating RBP4 as marker independent of obesity. Further analysis showed that the association between RBP4 and component 1 was limited only to women, and here the correlation remained significant even after adjusting the data for BMI. Thus, results from this study lend support to earlier suggestions that RBP4 may serve as an independent marker of CVD in women.

Several studies substantiate a link between RBP4 and CVD. Multiple regression analysis in a Japanese study revealed that serum RBP4 level was significantly related to systolic BP independently of age, sex, BMI, and total cholesterol level [19]; in another Japanese study, plasma RBP4 levels in the subjects with cerebral infarction were significantly greater than those in control subjects [20]. The present data show significantly higher systolic blood pressure in CVD compared to healthy control group as well as a close association between component 1 and systolic blood pressure in the CVD group. RBP4 levels were increased in hypertensive women and correlated with the degree of carotid intima-media thickness (IMT), suggesting a role for this adipokine in the modulation of the atherosclerotic process exerted by the adipose tissue [17]. A recent study found elevated serum RBP4 to be associated with small dense low-density lipoprotein cholesterol (sdLDL-C) and oxidized low-density lipoprotein (ox-LDL) levels in dyslipidemic subjects [30].

A previous study reported subcutaneous adipose tissue-derived RBP4-mRNA expression to be gender specific and higher in women [31]. Studies have also shown gender differences in the nature and frequency of CVD. Acute coronary syndrome with normal coronary arteries (non-obstructive CAD) were found to be more common in women (10–25%) when compared to men (6–10%) [32], [33] suggesting that, in women, atherosclerosis can occur with seemingly normal coronary arteries. Plaque erosion, compared with the classic plaque fissure and rupture, as the etiology of coronary thrombosis, was more frequent in women than in men (37 compared with 18%, respectively) [32], [33]. The gender bias in atherogenesis was attributed to effects of the sex steroids (oestrogens, androgens and progestins) on various cell types involved in the disease process [34]. In this context, correlation between RBP4 and CVD in women may be indicative of a causative link.

In our study, in addition to RBP4 various cardiometabolic risk factors like age, BMI, systolic blood pressure, triglyeride and HDL levels were significantly associated with CVD compared to healthy control group. However, a study involving 709 recent postmenopausal women showed only a weak correlation between elevated serum RBP4 and higher triglycerides levels, but no additional associations between RBP4 levels and other cardiometabolic risk factors [35]. In another study involving 473 subjects, RBP4 positively correlated with triglycerides but not with other cardiometabolic risk factors [28]. Our study did not show any association between serum LCN2 levels and CVD, even though LCN2 levels correlated significantly with FABP4 and RBP4 in the entire study population. Plasma LCN2 was found to be a significant predictor of CVD associated mortality, independent of traditional risk factors, in older adults [36].

Previous studies have associated serum RBP4 levels to obesity, insulin resistance and components of the metabolic syndrome [19], [21]. RBP4 level was positively associated with visceral fat and LDL-cholesterol levels, independently of body weight, in a study involving 102 women and the study authors suggested a causative link between visceral fat derived RBP4 and CVD in women [37]. Furthermore, RBP4 level was increased in serum of lean and obese insulin-resistant humans compared to insulin-sensitive humans, indicating that higher RBP4 may reflect insulin-resistance independently of obesity [8]. In another study, circulating RBP4 levels were independent of adipose tissue secreted RBP4, which is supported by the finding that RBP4 mRNA transcription in obese persons, as compared to lean subjects, increased 60-fold in visceral fat and only 12 fold in subcutaneous fat [38].

In our study, FABP4 levels in CVD subjects were significantly higher than in healthy controls. However, FABP4 levels in both obese diabetes and obese non-diabetes subjects also differed significantly from non-obese subjects confirming the previously reported specificity of FABP4 as a biomarker of adipose tissue mass. Hence, it is clear from the results that FABP4 level is not a marker differentiating CVD from obesity in the studied population.

There were some limitations to our study. Our study subjects consisted men and women of Saudi ethnicity, and hence the generalizability to other ethnicities is unknown. Even though several potential risk factors of CVD were included in our analysis, the role of some unknown or unmeasured confounders cannot be ruled out.

In summary, results of our study imply that the measurement of serum levels of RBP4 in women can be considered along with other cardiovascular risk factors in a larger clinical setting, as a valuable predictor of the risk of developing major cardiovascular events. Also, determining RBP4 levels in serum may help to improve understanding of the disease process, to obtain prognostic information, and to guide and follow the efficacy of cardiovascular therapies.

## Supporting Information

Table S1 (DOCX)
