# Supplementary material for: Serum Retinol-Binding Protein 4 as a Marker for Cardiovascular Disease in Women
Source: PLoS One. 2012 Oct 31;7(10):e48612. doi: 10.1371/journal.pone.0048612 (PMC3485358; doi:10.1371/journal.pone.0048612)
Supplement: Table S1 — (DOCX) [file pone.0048612.s001.docx]

**Table S1. Gender differences in the levels of serum RBP4, FABP4 and LCN2**

|  | **Overall** | | | **Healthy Control** | | | **Obese Without Diabetes** | | | **Non-obese Diabetes** | | | **Obese Diabetes** | | | **CVD** | | |
| --- | --- | --- | --- | --- | --- | --- | --- | --- | --- | --- | --- | --- | --- | --- | --- | --- | --- | --- |
|  | *Female* | *Male* | *p value* | *Female* | *Male* | *p value* | *Female* | *Male* | *p value* | *Female* | *Male* | *p*  *value* | *Female* | *Male* | *p value* | *Female* | *Male* | *p value* |
| RBP4 (ng/mL) | 4.9±0.36 | 6.1±0.45 | <0.001 | 4.7±0.37 | 6.1±0.31 | 0.002 | 4.4±0.38 | 6.1±0.50 | 0.003 | 5.2±0.33 | 6.1±0.39 | 0.05 | 5.0±0.35 | 5.4±0.34 | 0.39 | 5.2±0.40 | 7.2±0.65 | 0.04 |
| FABP4  (ng/mL) | 2.0±0.84 | 1.7±0.96 | 0.30 | 1.1±0.56 | 1.05±0.47 | 0.73 | 2.3±0.81 | 2.1±0.92 | 0.68 | 1.8±0.86 | 1.4±0.64 | 0.49 | 2.6±0.99 | 1.8±0.65 | 0.29 | 2.8±0.78 | 2.6±0.90 | 0.89 |
| LCN2  (ng/mL) | 27.9±3.2 | 33.7±4.3 | 0.02 | 29.5±3.5 | 35.2±2.4 | 0.26 | 27.1±2.1 | 35.0±2.7 | 0.07 | 25.3±2.5 | 32.2±4.5 | 0.18 | 27.3±3.0 | 29.8±4.2 | 0.63 | 33.5±7.1 | 36.2±8.4 | 0.79 |
